# Unmet needs of adults living with mucopolysaccharidosis II: data from the Hunter Outcome Survey

**DOI:** 10.1186/s13023-024-03464-8

**Published:** 2025-07-01

**Authors:** Joseph Muenzer, Hernan Amartino, Roberto Giugliani, Paul Harmatz, Shuan-Pei Lin, Bianca Link, David Molter, Uma Ramaswami, Maurizio Scarpa, Jaco Botha, Jennifer Audi, Barbara K. Burton

**Affiliations:** 1 University of North Carolina at Chapel Hill, Chapel Hill, NC 27514 USA; 2 Hospital Universitario Austral, Buenos Aires, Argentina; 3 Department of Genetics UFRGS, Medical Genetics Service HCPA, INAGEMP, DASA Genomica and Casa Dos Raros, Porto Alegre, Brazil; 4 UCSF Benioff Children’s Hospital Oakland, Oakland, CA USA; 5 Mackay Memorial Hospital, Taipei, Taiwan; 6 University Children’s Hospital, Zurich, Switzerland; 7 Washington University School of Medicine, St. Louis, MO USA; 8 Lysosomal Storage Disorders Unit, Royal Free London NHS Foundation Trust, London, UK; 9 Udine University Hospital, Udine, Italy; 10 Takeda Pharmaceuticals International AG, Zurich, Switzerland; 11 Ann & Robert H Lurie Children’s Hospital of Chicago, Northwestern University, Chicago, IL USA; 12 Present Address: Takeda Pharmaceuticals International AG, Ultragenyx Europe GmbH, Lichstrasse, Basel, Switzerland

**Keywords:** Adults, Cognitive Impairment, Hunter Syndrome, Mucopolysaccharidosis type II, Neuronopathic, Non-neuronopathic

## Abstract

**Background:**

Mucopolysaccharidosis II (MPS II) is a rare, life-limiting lysosomal storage disease caused by deficient iduronate-2-sulfatase activity. The current standard of care for MPS II is intravenous enzyme replacement therapy (ERT), which has been shown to improve somatic signs and symptoms and to increase life expectancy by approximately 12 years. This study reported on the somatic disease burden and clinical requirements of adult male patients in the Hunter Outcome Survey (ClinicalTrials.gov Identifier: NCT03292887).

**Results:**

Of the 373 patients in the analysis, 88 (23.6%) had cognitive impairment and 332 (89.0%) had received ERT. Almost half of all ERT-treated patients (47.0%) had undergone surgery in adulthood; the most common surgery was hernia repair (17.8% of patients). Over one-third (38.6%) reported hearing aid use. The median 6-min walk test distance for 151 treated patients was 436.0 m at the latest assessment after 18 years of age. Cardiovascular signs and symptoms were present in 71.6% (192/268) of patients and 27.3% (60/220) reported oxygen dependency after 18 years of age. Approximately half (50.9%) of ERT-treated patients experienced at least one serious adverse event in adulthood, with the most common being respiratory disorders. Intravenous ERT was well tolerated, with a rate of serious infusion-related reactions in adulthood of 0.03 per 10 patient-years.

**Conclusions:**

Overall, adult patients with neuronopathic and non-neuronopathic MPS II had a high disease burden and requirement for surgeries, emphasizing the need to continue multidisciplinary management and regular assessments in adulthood. Further research into the differences in care needs of adult patients with MPS II is warranted.

*Trial registration* NCT03292887.

**Supplementary Information:**

The online version contains supplementary material available at 10.1186/s13023-024-03464-8.

## BACKGROUND

Mucopolysaccharidosis II (MPS II; Hunter syndrome; OMIM 309900) is a rare, progressive, X-linked lysosomal storage disease caused by deficient activity of iduronate-2-sulfatase (I2S), which is responsible for the degradation of the glycosaminoglycans (GAGs) dermatan sulfate and heparan sulfate [1, 2]. The deficiency of I2S activity results in the accumulation of GAGs throughout the body, leading to a diverse range of signs and symptoms that typically present at 2–4 years of age [1, 2]. Somatic manifestations include coarse facial features, hepatosplenomegaly, inguinal and umbilical hernias, respiratory disease, cardiovascular disease, and joint stiffness associated with restricted movement [2–5]. Around two-thirds of patients also have central nervous system involvement (neuronopathic disease) and experience cognitive impairment and developmental and/or speech delays and decline [6, 7]. These patients rarely survive beyond the second decade of life; patients with non-neuronopathic disease typically survive longer [8, 9].

Enzyme replacement therapy (ERT) with intravenous recombinant I2S (idursulfase [marketed as Elaprase]; Takeda Pharmaceuticals U.S.A., Inc., Lexington, MA, USA) is the current standard of care for MPS II and has been shown to improve and/or stabilize somatic manifestations in pediatric and adult patients, including mobility, respiratory function, hepatosplenomegaly, and cardiac disease [10–18]. Owing to its inability to cross the blood–brain barrier, intravenous idursulfase is not expected to treat the cognitive signs and symptoms of MPS II [10, 19, 20].

With the availability of intravenous idursulfase, patients with MPS II are surviving longer and increasingly reaching adulthood [21]. Treated patients are reported to have a 54% lower risk of death than untreated patients, with 11.8 additional years of median survival compared with untreated patients (33.0 years vs. 21.2 years, respectively; median treatment onset 7 years old) [21]. Consistent with the progressive nature of MPS II, the clinical characteristics and, therefore, management requirements, differ between adult and pediatric patients. As patients age, there is a reduction in joint mobility caused by progressive joint contractures, an increase in the risk and severity of cardiac hypertrophy and valvular heart disease, an increase in the risk of airway obstruction and collapse, and an increase in airway management difficulties during anesthesia [22–27]. Airway management during anesthesia is important in patients with MPS II given the frequent requirement for surgical interventions [23, 25, 28]. To date, few studies on MPS II have been conducted specifically in patients over 18 years of age [29–31]. Therefore, there is a need to increase our understanding of the clinical characteristics of adult patients with MPS II and the implications for optimizing management and support.

The Hunter Outcome Survey (HOS), a global, multicenter, observational registry founded in 2005 and funded by Takeda, collected long-term data on the natural history of MPS II and the effects of intravenous idursulfase [3]. Global registries offer the opportunity to follow a diverse range of patients over a long period of time and are useful in the context of rare diseases, in which patient numbers are small. The present study aims to assess the somatic disease burden and clinical requirements of adult patients with neuronopathic and non-neuronopathic MPS II in HOS, to help to inform clinical management and improve the quality of life of adult patients.

## Methods

### HOS registry design

HOS (ClinicalTrials.gov Identifier: NCT03292887) was designed to collect a broad range of data on patients with MPS II, including data on vital signs, laboratory assessments, signs and symptoms of MPS II, and treatment; data were obtained during routine patient visits and assessments [3]. Eligible patients had a biochemically or genetically confirmed diagnosis of MPS II and could be untreated, could be receiving treatment with intravenous idursulfase, or could have undergone a bone marrow transplant; patients receiving any other form of ERT or enrolled in an interventional clinical trial were excluded. Data could be collected for patients who were alive at the point of enrollment (prospective patients) or deceased before enrollment (retrospective patients) if local regulations permitted. Before patient enrollment, Independent Review Board/Ethics Committee approval was obtained for all participating centers. Written informed consent was also obtained from each patient, their parents, or a legal representative. All patient information is managed in accordance with national data protection standards.

The presence of cognitive impairment was recorded in HOS based on the answer to the following question: “Cognitive impairment? Yes/No”. This may be informed by the clinical impression of the healthcare professional or by formal cognitive tests.

### Patient population

As of January 23, 2023, 1405 patients were enrolled in HOS from 108 centers in 29 countries. Of these, 1235 patients were followed prospectively and 170 had data entered retrospectively. The population of the present analysis comprised all male patients enrolled in HOS who were aged 18 years or older at their latest available visit as of January 23, 2023; these patients were enrolled between October 28, 2005 and July 27, 2022. Adult female patients (*n* = 3) were excluded because they are rare and may have a different disease progression from male patients owing to the X-linked nature of MPS II. Patients who had received a bone marrow transplant were also excluded.

### Data analysis

Demographic data, surgeries, and safety data were summarized for all patients in the analysis population who had the relevant information available at the data cut-off.

The number and types of surgeries conducted in patients after the age of 18 years were analyzed for treated and untreated patients. Treated patients were defined as those who had received at least one dose of idursulfase and who were still receiving treatment when aged 18 years or older. Surgeries entered into the ‘other’ category in the HOS database were reclassified into existing categories by the HOS medical monitor based on descriptions in the free-text field.

Urinary GAGs (uGAGs), 6-min walk test (6MWT), left ventricular mass index (LVMI), forced vital capacity (FVC), and forced expiratory volume in 1 s (FEV_1_) were analyzed at baseline and latest visits for all patients with available data. For these analyses, first and latest visits may have occurred before or after the age of 18 years. uGAG levels recorded in HOS were obtained from, and determined by, methods used in either a local or a central laboratory; it should be noted that the methods and reference ranges used are likely to have differed between laboratories. For the analysis of 6MWT, FVC, and FEV_1_ assessments over time, patients were excluded if the baseline assessments were completed when the patient was younger than 5 years of age or if they required assistance to complete the test. FVC and FEV_1_ were expressed as a percentage of predicted values, with reference to the expected value in an individual without MPS II of the same age, sex, and height [32, 33]. LVMI was calculated from an echocardiogram using the Devereux formula and adjusted for body surface area using the closest height and weight measurements taken within 91.5 days either side of the assessment date [34]. LVMI values above 400 g/m^2^ were considered to be unreliable and were excluded.

The clinical parameters described above, and cardiovascular signs and symptoms, were also analyzed at the latest visit after 18 years of age in the subgroup of patients treated with idursulfase. Both absolute values and percentage of predicted values were assessed for FVC and FEV_1_. Untreated patients were not analyzed owing to the small number of patients in this group (*n* = 41). The presence or absence of cardiovascular signs and symptoms was inferred from the latest entries in the HOS ‘signs and symptoms’ forms.

Serious adverse events (SAEs) and infusion-related reactions (IRRs) were analyzed in patients treated with idursulfase and calculated as rates per 10 patient-years (PY) of follow-up. Events were coded as either infusion-related or not infusion-related, with infusion-related events being defined as events occurring during or within 24 h of an infusion and with evidence of a causal relationship with idursulfase. SAEs and IRRs were excluded from the analyses in cases when there was a missing start date. Causes of death not covered by the main database fields were recorded as ‘other’, detailed using free text, and then reviewed for reclassification by the HOS medical monitor.

All data are summarized with descriptive statistics; median values (10th percentile [P10], 90th [P90] percentile) are provided here, unless stated otherwise. Data were also analyzed by the absence or presence of cognitive impairment at the latest visit. Patients with cognitive impairment at the latest visit were included in the neuronopathic group; all other patients were included in the non-neuronopathic group. All available data were assessed; no age limit was applied to the collection of data on clinical manifestations and surgeries in the two groups.

## Results

**Fig. 1:**
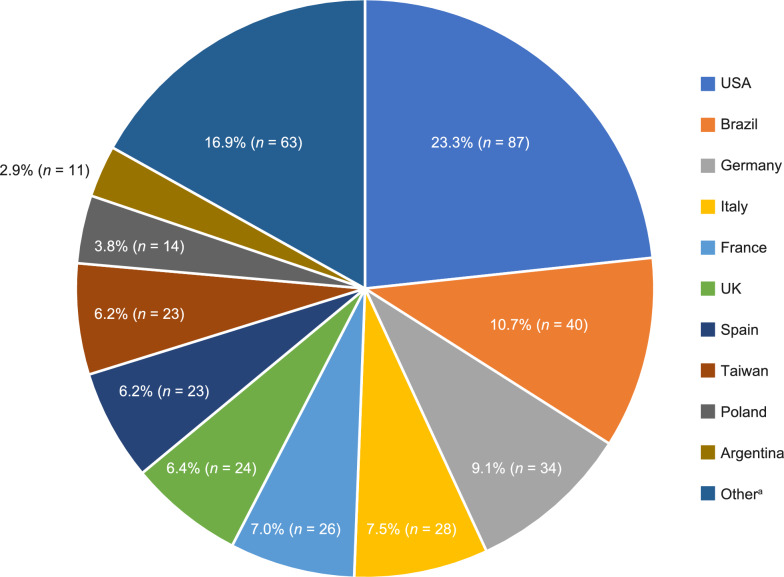
Location of adult patients in HOS included in this analysis (*N* = 373). ^a^Other countries (< 10 patients each) included: Austria, Belgium, Bulgaria, Canada, Chile, Colombia, Croatia, Czechia, Denmark, Greece, Ireland, Japan, Netherlands, Norway, Portugal, Romania, Sweden, Switzerland, and Venezuela. *Abbreviations*: HOS, Hunter Outcome Survey

In total, 373 males from across 29 countries were included in this analysis, 88 of whom had cognitive impairment (neuronopathic MPS II) at the latest visit and 269 of whom had no cognitive impairment (non-neuronopathic MPS II) at the latest visit (Fig. 1; Table 1); cognitive data were not available for 16 patients. Overall, the countries with the highest numbers of patients were the USA (87/373, 23.3%), Brazil (40/373, 10.7%), and Germany (34/373, 9.1%). In patients with neuronopathic and non-neuronopathic MPS II, respectively, median (P10, P90) age at symptom onset was 2.0 (0.3, 4.8) years and 2.5 (0.6, 7.0) years, median age at diagnosis was 4.0 (2.0, 7.0) years and 5.0 (1.5, 14.5) years, and median age at the latest visit was 21.3 (18.6, 30.5) years and 25.4 (18.9, 43.4) years. Among patients with neuronopathic MPS II, 65.9% were aged 18 years to less than 23 years and 85.2% were aged 18 years to less than 28 years at the latest visit compared with 36.9% and 57.6% of patients with non-neuronopathic disease, respectively (Additional file 1: Fig. S1).

**Table 1:** Patient characteristics (*N* = 373)

	Neuronopathic (*n* = 88)	Non-neuronopathic (*n* = 269)	All patients (*N* = 373)^a^
Age at first symptom onset, years	*n* = 82	*n* = 220	*n* = 308
Median (P10, P90)	2.0 (0.3, 4.8)	2.5 (0.6, 7.0)	2.0 (0.5, 6.0)
Age at diagnosis, years	*n* = 86	*n* = 254	*n* = 348
Median (P10, P90)	4.0 (2.0, 7.0)	5.0 (1.5, 14.5)	4.6 (1.5, 14.0)
Age at HOS entry, years	*n* = 88	*n* = 269	*n* = 373
Median (P10, P90)	13.2 (5.9, 20.9)	18.1 (8.2, 35.6)	17.3 (7.3, 33.2)
Time in HOS, years	*n* = 88	*n* = 269	*n* = 373
Median (P10, P90)	10.9 (2.3, 15.1)	8.1 (1.0, 14.9)	8.2 (0.8, 14.9)
Time in HOS as an adult, years	*n* = 88	*n* = 269	*n* = 373
Median (P10, P90)	3.3 (0.6, 12.5)	7.4 (0.9, 25.4)	6.2 (0.8, 21.7)
Age at latest visit, years	*n* = 88	*n* = 269	*n* = 373
Median (P10, P90)	21.3 (18.6, 30.5)	25.4 (18.9, 43.4)	24.2 (18.8, 39.7)
Cognitive impairment, *n* (%)	*n* = 88	*n* = 269	*n* = 357
At any time	88 (100.0)	74 (27.5)	162 (45.4)
At latest visit	88 (100.0)	0 (0.0)	88 (24.6)
Age at start of idursulfase treatment, years	*n* = 78	*n* = 243	*n* = 332
Median (P10, P90)	11.1 (5.5, 21.0)	15.8 (6.8, 32.8)	14.9 (6.3, 30.9)
Duration of idursulfase treatment, years	*n* = 78	*n* = 243	*n* = 332
Median (P10, P90)	12.0 (2.8, 15.6)	11.0 (2.3, 15.7)	11.0 (2.1, 15.6)
Deceased, *n* (%)	45 (51.1)	72 (26.8)	120 (32.2)
Age at death, years	*n* = 45	*n* = 72	*n* = 120
Median (P10, P90)	20.8 (18.6, 29.0)	24.9 (18.9, 42.2)	22.7 (18.7, 36.3)

^a^Cognitive data were not available for some patients; the number of patients in the ‘all patients’ population is therefore not equal to the combined total of the neuronopathic and non-neuronopathic MPS II populations

*HOS* Hunter Outcome Survey, *MPS II* mucopolysaccharidosis II, *P10* 10th percentile, *P90* 90th percentile

The median (P10, P90) age at HOS entry was 13.2 (5.9, 20.9) years for patients with neuronopathic MPS II and 18.1 (8.2, 35.6) years for patients with non-neuronopathic MPS II, the median (P10, P90) follow-up time after the age of 18 years was 3.3 (0.6, 12.5) years and 7.4 (0.9, 25.4) years, respectively.

Most patients (332/373, 89.0%) had received at least one dose of idursulfase, with similar proportions of patients with neuronopathic and non-neuronopathic MPS II treated (88.6% and 90.3%, respectively). The median (P10, P90) age at the start of idursulfase treatment was 11.1 (5.5, 21.0) years and 15.8 (6.8, 32.8) years in patients with neuronopathic and non-neuronopathic MPS II, respectively, and the median duration of idursulfase treatment was similar in the two groups (12.0 [2.8, 15.6] years and 11.0 [2.3, 15.7] years, respectively).

Patient characteristics in the treated (*n* = 332) and the untreated (*n* = 41) populations are shown in Additional file 1: Table S1. Notable differences included the median (P10, P90) time in HOS (treated patients: 9.0 [1.5, 15.1] years; untreated patients: 2.2 [0.0, 8.4] years) and the median (P10, P90) age at HOS entry (treated patients: 16.7 [7.0, 32.1] years; untreated patients: 21.0 [12.6, 48.3] years). The median age at diagnosis and the median age at symptom onset were similar in the treated and untreated populations, as were the proportions of deceased patients and the proportions of patients with cognitive impairment.

Overall, 144/373 patients (38.6%) reported the use of a hearing aid with a known start date; the median age of hearing aid first use was 7.7 (3.6, 18.2) years, and 88.2% of these patients had a hearing aid in place before the age of 18 years. These findings were similar regardless of patient cognitive impairment status.

### Surgeries

Almost half of all treated patients (156/332, 47.0%) had undergone at least one surgery when aged 18 years or older, with 416 surgeries in total. Most patients who had surgery underwent one (59/156, 37.8%) or two (45/156, 28.8%) different surgical procedures (median [P10, P90] number of surgeries, 2 [1, 6]), although a higher requirement for surgery was reported in some individuals, with one patient undergoing 17 different surgeries. Of the 156 treated patients who had undergone surgery, more than one-third required repeat surgery of the same type. Hernia repair, carpal tunnel decompression, and port-a-cath placement/replacement were repeated in more than 10 patients (Additional file 1: Table S2).

The proportion of treated patients who had undergone at least one surgery aged 18 years or older was 29.5% (23/78) in the neuronopathic group and 54.3% (132/243) in the non-neuronopathic group; the median (P10, P90) number of different types of surgical procedures per patient was the same in the neuronopathic group and in the non-neuronopathic group (2 [1, 5] and 2 [1, 6], respectively). Notably, 77.4% (257/332) of treated adult patients had undergone at least one surgery before the age of 18 years old. Hernia repair, which was reported in 47.6% of treated patients, was the most frequently performed surgery during childhood (Additional file 1: Table S3).

In total, 11 untreated patients (11/41, 26.8%), all in the non-neuronopathic group (11/26, 42.3%), had undergone surgery as an adult, with 25 surgeries performed in total. The median number (P10, P90) of different types of surgery per patient was 2 (1, 5) and the maximum was 6. Just over half of the 41 untreated patients (21/41, 51.2%) also underwent one or more surgeries before the age of 18 years.

**Fig. 2:**
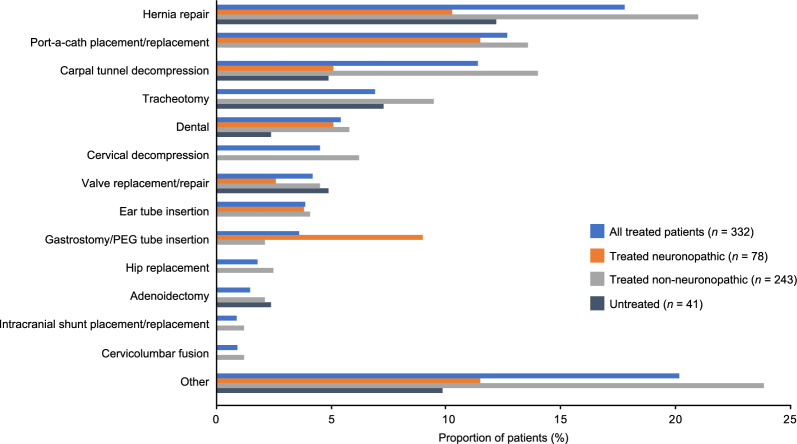
Proportions of patients who received specific surgeries (conducted in ≥ 1% of adult treated patients). Data are shown for treated patients with neuronopathic and non-neuronopathic MPS II, and untreated patients. All untreated patients who had at least one surgery had non-neuronopathic MPS II. Port-a-cath placement/replacement is a treatment-related procedure. The ‘other’ category included Achilles lengthening, cervical fusion, genu varum, hip osteotomy, femoral osteotomy, knee arthroscopy, tonsillectomy, and other surgeries that did not fit into the predefined HOS categories. Reclassification of ‘other’ surgeries is described in Additional file 1: Table S7. *Abbreviations*: HOS, Hunter Outcome Survey; MPS II, mucopolysaccharidosis II; PEG, percutaneous endoscopic gastrostomy

**Fig. 3:**
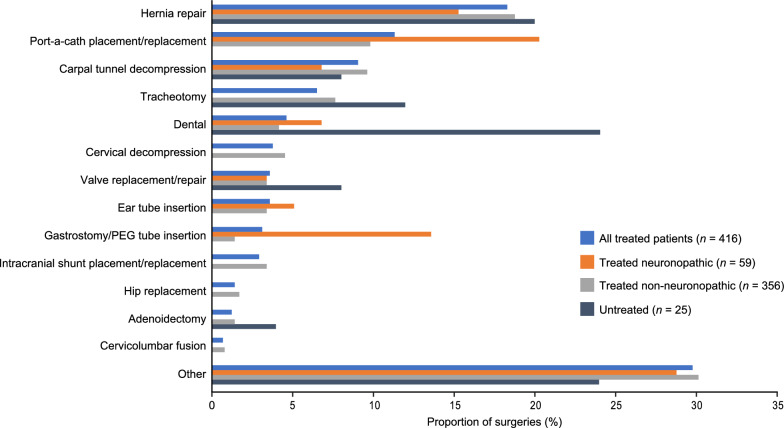
Specific surgeries as proportions of all surgeries (representing ≥ 1% of all surgeries in adult treated patients). Data are shown for treated patients with neuronopathic and non-neuronopathic MPS II, and untreated patients. All untreated patients who had at least one surgery had non-neuronopathic MPS II. Port-a-cath placement/replacement is a treatment-related procedure. The ‘other’ category included Achilles lengthening, cervical fusion, genu varum, hip osteotomy, femoral osteotomy, knee arthroscopy, tonsillectomy, and other surgeries that did not fit into the predefined HOS categories. *Abbreviations*: HOS, Hunter Outcome Survey; MPS II, mucopolysaccharidosis II; PEG, percutaneous endoscopic gastrostomy

The surgery performed in the highest proportion of adult patients was hernia repair, which was performed in 17.8% of treated patients (59/332) and 12.2% of untreated patients (5/41; Fig. 2). Hernia repair was also the most common surgery in treated patients, representing 76 out of 416 (18.3%) surgical procedures. In untreated patients, the most common surgery was dental followed by hernia repair (24% and 20% of surgical procedures, respectively) (Fig. 3).

Among adult treated patients, hernia repair, carpal tunnel decompression, tracheotomy, and cervical decompression were reported in a higher proportion of patients with non-neuronopathic than with neuronopathic MPS II (more than 5% difference). No patients with neuronopathic MPS II underwent tracheotomy or cervical decompression after the age of 18 years (Fig. 2). Of those with available data, the proportion of patients who underwent surgeries before the age of 18 years was similar in the patients with non-neuronopathic and neuronopathic MPS II (hernia repair, 114/187 [61.0%] and 44/69 [63.8%]; carpal tunnel decompression, 74/187 [39.6%] and 22/69 [31.9%]; tracheotomy, 7/187 [3.7%] and 3/69 [4.3%]; cervical decompression, 6/187 [3.2%] and 3/69 [4.3%], respectively).

After the age of 18 years, the proportion of treated patients who had undergone gastrostomy/percutaneous endoscopic gastrostomy (PEG) tube insertion was higher (more than 5% difference) for patients with neuronopathic than with non-neuronopathic MPS II. Port-a-cath placement/replacement, dental, valve replacement/repair, and ear tube insertion surgeries were carried out in similar proportions of treated patients with neuronopathic and non-neuronopathic MPS II (less than 3% difference). When the numbers of individual surgeries were considered (Fig. 3), hernia repair represented a similar proportion of all surgeries in patients with treated neuronopathic and non-neuronopathic MPS II (15.3% and 18.8%, respectively), and port-a-cath placement/replacement represented a higher proportion of all surgeries in treated patients with neuronopathic versus non-neuronopathic MPS II (20.3% vs. 9.8%). Of the patients who had undergone tracheotomy or gastrostomy procedures and who were deceased (*n* = 16), three died within 1 month of their procedure (two who had received tracheotomies [one with inguinal hernia repair within the same month] and one patient with removal of obstructing granulation tissue and tracheostomy tube change), and one died within 2 months of their surgical procedures (tracheotomy and cervical laminectomy within the same month). One other patient died within 1 month of an inguinal hernia repair.

**Fig. 4:**
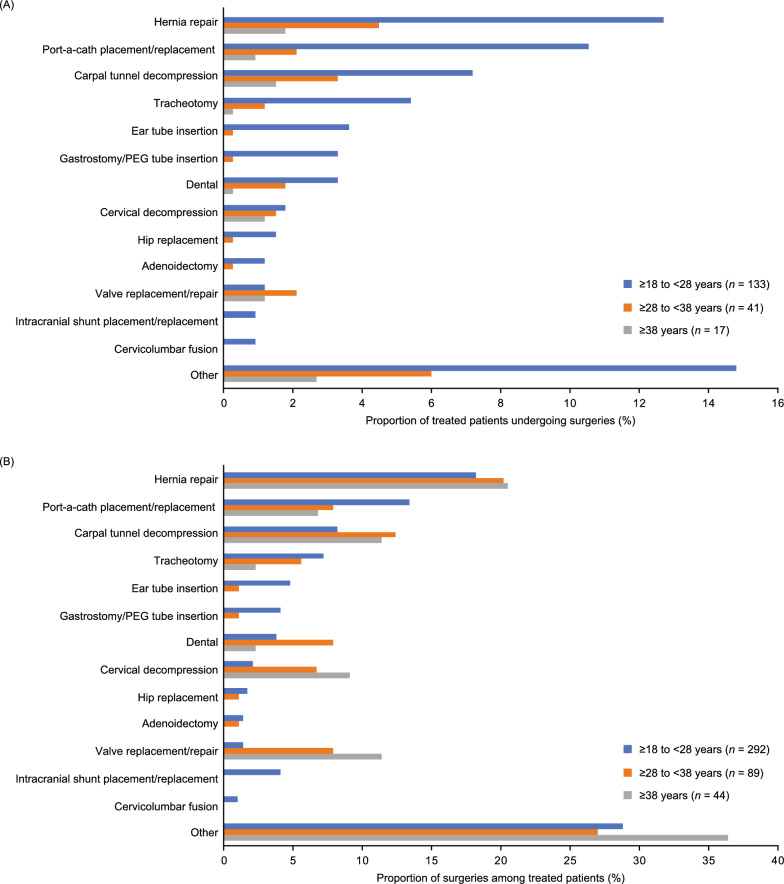
Surgeries in treated patients by age category by (**A**) proportion of patients and (**B**) proportion of surgeries. The ‘other’ category included Achilles lengthening, cervical fusion, genu varum, hip osteotomy, femoral osteotomy, knee arthroscopy, tonsillectomy, and other surgeries that did not fit into the predefined HOS categories. *Abbreviations*: HOS, Hunter Outcome Survey; PEG, percutaneous endoscopic gastrostomy

Surgeries conducted in the highest proportion of treated patients aged from 18 to 27 years and aged from 28 to 37 years were hernia repair (12.7% and 4.5%, respectively) and port-a-cath placement/replacement (10.5% and 2.1%, respectively). Among treated patients aged 38 years and over, surgeries performed in the most patients were hernia repair (1.8%) and carpal tunnel decompression (1.5%) (Fig. 4A). Similar trends were observed when the individual surgeries were considered as a proportion of all surgeries (Fig. 4B).

### Clinical parameters

In treated patients, the median (P10, P90) uGAG level was 23.5 (4.3, 198.2) μg/mg creatinine and the 6MWT distance was 436.0 (214.0, 600.0) m at the latest assessment after 18 years of age, with median (P10, P90) ages at the latest assessment of each parameter of 23.4 (18.7, 41.7) years and 26.2 (19.4, 40.3) years, respectively (Table 2). Five patients were unable to complete the 6MWT without assistance and so were excluded. Out of 268 patients with available data, 192 (71.6%) had cardiovascular signs and symptoms at their latest visit, and the most reported cardiovascular abnormality was valve disease, which was documented in 182/266 patients (68.4%). The median (P10, P90) percentages of predicted FVC and FEV_1_ were 59.4% (35.0%, 85.3%) and 46.8% (23.1%, 74.7%), respectively, with a median age at latest assessment of both parameters of around 27 years (Table 2). Of 220 patients with available data, over one-quarter (60, 27.3%) reported oxygen dependency after 18 years of age.

**Table 2:** Clinical parameters in treated patients at latest assessment after age 18 years (*N* = 332)

	Neuronopathic (*n* = 78)	Non-neuronopathic (*n* = 243)	All patients (*N* = 332)^a^
uGAG	*n* = 3	*n* = 25	*n* = 28
Mean (SD), μg/mg creatinine	104.3 (103.0)	56.7 (96.2)	61.8 (96.1)
Median (P10, P90), μg/mg creatinine	87.3 (10.9, 214.8)	10.9 (4.3, 120.0)	23.5 (4.3, 198.2)
Age at latest assessment, median (P10, P90) years	22.4 (21.4, 30.5)	23.5 (18.7, 41.7)	23.4 (18.7, 41.7)
6MWT	*n* = 23	*n* = 127	*n* = 151
Mean (SD), m	294.9 (137.6)	434.3 (157.5)	413.5 (161.9)
Median (P10, P90), m	306.0 (84.0, 462.0)	451.0 (249.0, 610.0)	436.0 (214.0, 600.0)
Age at latest assessment, median (P10, P90) years	23.6 (20.7, 32.5)	26.8 (19.2, 41.7)	26.2 (19.4, 40.3)
Any cardiovascular signs and symptoms			
*n/N* (%)	48/62 (77.4)	144/205 (70.2)	192/268 (71.6)
Valve disease			
*n/N* (%)	46/61 (75.4)	136/204 (66.7)	182/266 (68.4)
Cardiomyopathy			
*n/N* (%)	7/61 (11.5)	17/203 (8.4)	24/265 (9.1)
Arrhythmia/palpitations			
*n/N* (%)	3/61 (4.9)	19/195 (9.7)	22/257 (8.6)
LVMI^b^	*n* = 25	*n* = 103	*n* = 128
Mean (SD), g/m^2^	87 (43)	99 (38.9)	97 (39.8)
Median (P10, P90), g/m^2^	78 (59, 110)	90 (62, 152)	88 (59, 151)
Age at latest assessment, median (P10, P90) years	22.2 (18.7, 29.2)	26.8 (19.2, 41.0)	24.3 (18.9, 39.3)
Absolute FVC	*n* = 18	*n* = 145	*n* = 164
Mean (SD), L	3.3 (6.0)	2.8 (4.7)	2.8 (4.8)
Median (P10, P90), L	1.8 (0.9, 4.2)	2.3 (1.1, 4.1)	2.2 (1.1, 4.1)
Age at latest assessment, median (P10, P90) years	27.9 (19.1, 35.0)	27.2 (19.8, 42.5)	27.1 (19.7, 41.9)
Percentage of predicted FVC	*n* = 18	*n* = 143	*n* = 162
Mean (SD), %	55.0 (21.2)	61.1 (19.7)	60.4 (19.8)
Median (P10, P90), %	49.5 (31.8, 96.5)	62.0 (35.0, 85.0)	59.4 (35.0, 85.3)
Age at latest assessment, median (P10, P90) years	25.5 (19.1, 35.0)	26.8 (19.5, 42.5)	26.7 (19.5, 41.9)
Absolute FEV_1_	*n* = 18	*n* = 145	*n* = 164
Mean (SD), L	2.7 (5.2)	1.9 (2.3)	2.0 (2.7)
Median (P10, P90), L	1.3 (0.5, 3.5)	1.6 (0.6, 3.3)	1.5 (0.6, 3.3)
Age at latest assessment, median (P10, P90) years	27.9 (19.1, 35.0)	27.2 (19.8, 42.5)	27.1 (19.7, 41.9)
Percentage of predicted FEV_1_	*n* = 18	*n* = 142	*n* = 161
Mean (SD), %	42.4 (20.8)	49.0 (20.2)	48.2 (20.2)
Median (P10, P90), %	39.0 (19.0, 84.9)	47.0 (24.0, 74.7)	46.8 (23.1, 74.7)
Age at latest assessment, median (P10, P90) years	25.5 (19.1, 35.0)	26.9 (19.7, 42.5)	26.8 (19.6, 41.9)

^a^Cognitive data were not available for some patients; the number of patients in the ‘all patients’ population is therefore not equal to the combined total of the neuronopathic and non-neuronopathic MPS II populations

^b^Three last assessment LVMI values were excluded from the data set because they were greater than 400 g/m^2^ and were assumed to be data entry errors

*6MWT* 6-min walk test, *FEV*_1_ forced expiratory volume in 1 s, *FVC* forced vital capacity, *LVMI* left ventricular mass index, *m* meters, *MPS II* mucopolysaccharidosis II, *P10* 10th percentile, *P90* 90th percentile, *SD* standard deviation, *uGAG* urinary glycosaminoglycan

Limited data were available to explore the impact of age at treatment start on clinical parameters; however, patients who started treatment before 9 years of age tended to have higher 6MWT distances and lower rates of cardiomyopathy and arrhythmia or palpitations than those who started treatment later in life (Additional file 1: Table S4).

Overall clinical parameters for all patients at first and latest assessments, summarizing data collected before and after the age of 18 years, are shown in Additional file 1: Table S5.

### Adverse events

Out of 332 treated patients, 169 (50.9%) experienced at least one SAE when aged 18 years or older (3.0 SAEs per 10 PY of follow-up); of the 220 patients with available adverse event data, 87 (39.5%) experienced an SAE before the age of 18 years (1.8 SAEs per 10 PY). SAEs resulting from infections and infestations were common in adult treated patients (155 events reported in 68 patients; Table 3). Pneumonia was the most common SAE resulting from infections and infestations, occurring in more than 10.0% of patients (66 events reported in 38 patients). SAEs resulting from respiratory, thoracic and mediastinal disorders affected 21.7% of treated patients over the age of 18 years, with respiratory failure being the most frequently reported respiratory-related SAE (27 events in 25 patients). Overall, SAE rates after the age of 18 years were higher in patients with neuronopathic MPS II (3.8 SAEs per 10 PY) than in patients with non-neuronopathic MPS II (2.9 SAEs per 10 PY).

**Table 3:** Summary of SAEs reported in patients after age 18 years (*N* = 332)

	Treated, *n* (%)	
	Patients (*n* = 332)	Events (*n* = 553)
Any system organ class	169 (50.9)	553 (100.0)
Blood and lymphatic system disorders	1 (0.3)	1 (0.2)
Cardiac disorders	34 (10.2)	59 (10.7)
Cardiac arrest	11 (3.3)	11 (2.0)
Cardiac failure	5 (1.5)	5 (0.9)
Congenital, familial and genetic disorders	3 (0.9)	3 (0.5)
Ear and labyrinth disorders	3 (0.9)	3 (0.5)
Eye disorders	2 (0.6)	3 (0.5)
Gastrointestinal disorders	26 (7.8)	35 (6.3)
Inguinal hernia	5 (1.5)	7 (1.3)
Umbilical hernia	5 (1.5)	5 (0.9)
General disorders and administration site conditions	35 (10.5)	41 (7.4)
Death	19 (5.7)	19 (3.4)
Hepatobiliary disorders	1 (0.3)	1 (0.2)
Immune system disorders	3 (0.9)	3 (0.5)
Infections and infestations	68 (20.5)	155 (28.0)
Influenza	5 (1.5)	5 (0.9)
Pneumonia	38 (11.4)	66 (11.9)
Sepsis	6 (1.8)	6 (1.1)
Injury, poisoning and procedural complications	13 (3.9)	18 (3.3)
Investigations^a^	3 (0.9)	3 (0.5)
Metabolism and nutrition disorders	9 (2.7)	15 (2.7)
Musculoskeletal and connective tissue disorders	15 (4.5)	18 (3.3)
Cervical spine stenosis	7 (2.1)	7 (1.3)
Nervous system disorders	27 (8.1)	37 (6.7)
Carpal tunnel syndrome	8 (2.4)	12 (2.2)
Product issues^b^	5 (1.5)	9 (1.6)
Psychiatric disorders	4 (1.2)	4 (0.7)
Renal and urinary disorders	3 (0.9)	4 (0.7)
Respiratory, thoracic and mediastinal disorders	72 (21.7)	121 (21.9)
Acute respiratory failure	8 (2.4)	11 (2.0)
Dyspnea	9 (2.7)	10 (1.8)
Respiratory distress	10 (3.0)	11 (2.0)
Respiratory failure	25 (7.5)	27 (4.9)
Tracheomalacia	5 (1.5)	5 (0.9)
Skin and subcutaneous disorders	3 (0.9)	6 (1.1)
Vascular disorders	13 (3.9)	13 (2.4)

Data shown are total numbers of event per system organ class category and SAEs reported in five or more patients. Five SAEs from four patients were excluded owing to a missing start date and one death occurred that was not coded

^a^Investigations include blood creatinine increase, hemoglobin decrease, and oxygen saturation decrease

^b^Product issues include port-a-cath device failure, loosening, malfunction, or occlusion

*SAE* serious adverse event

In total, seven out of 332 patients (2.1%) receiving idursulfase had experienced nine serious IRRs when aged 18 years or older (0.03 serious IRRs per 10 PY). Serious IRRs were similar in patients with neuronopathic (0.06 serious IRRs per 10 PY) and patients with non-neuronopathic MPS II (0.05 serious IRRs per 10 PY). In patients aged 18 years or older, two of the nine serious IRRs occurred from 12 to 24 months after the start of ERT and seven occurred more than 24 months after the start of ERT. Idursulfase antibody status was unavailable for six of the nine serious IRRs that occurred more than 12 months after ERT was started. Of the three IRRs for which data were available, two patients tested positive for idursulfase antibodies, and one patient tested negative (all three events occurred more than 24 months after ERT was started).

At the time of this analysis, 45 out of 88 patients with neuronopathic MPS II (51.1%) and 72 out of 269 patients with non-neuronopathic MPS II (26.8%) had died, with a median (P10, P90) age at death of 20.8 (18.6, 29.0) years and 24.9 (18.9, 42.2) years, respectively. Overall, the most common known cause of death was respiratory failure (32/120, 26.7%), followed by cardiac arrest (13/120, 10.8%), and cardiorespiratory failure (12/120, 10.0%); these were also the top three most common known causes of death for patients with non-neuronopathic MPS II. For patients with neuronopathic MPS II, sepsis (4/45, 8.9%) was a more common cause of death than cardiac arrest (2/45, 4.4%) and cardiorespiratory failure (3/45 6.7%). Cause of death was unknown for 35/120 patients overall (29.2%). For treated patients, 73/104 (70.2%) of deaths occurred from the age of 18 years up to 28 years, 23/104 (22.1%) occurred from the age of 28 years up to 38 years, and 8/104 (7.7%) occurred in patients aged 38 years or older. Median survival based on Kaplan–Meier estimates (95% confidence intervals) in the treated population was 39.9 (34.5–48.3) years (Additional file 1: Fig. S2). The median age (P10, P90) at death was 23.0 (18.8, 36.1) years. Causes of death by age are presented in Additional file 1: Table S6.

## Discussion

This study provides the first in-depth analysis of exclusively adult patients with MPS II who are enrolled in HOS. Most enrolled patients were treated with idursulfase, and around one-quarter had cognitive impairment. Among treated patients, around half had undergone one or more surgeries, and more than one-third of patients used hearing devices. Hernia repair was the most common surgery overall, and the proportions of treated patients who underwent specific surgeries differed between patients with neuronopathic and patients with non-neuronopathic MPS II. Cardiovascular manifestations were present in approximately three-quarters of treated adult patients, and respiratory function was generally impaired. The most common known causes of death in patients with non-neuronopathic MPS II were related to respiratory or cardiac function, consistent with previous reports in the overall HOS population [16]; in patients with neuronopathic MPS II, sepsis was also a relatively common cause of death.

The age at symptom onset for patients with neuronopathic and non-neuronopathic MPS II (median of 2.0 years and 2.5 years, respectively) was slightly higher than what has previously been reported for pediatric and adult patients in HOS. Similarly, the age at diagnosis (median of 4.0 years for patients with neuronopathic MPS II and 5.0 years for patients with non-neuronopathic MPS II) was around 1 year older than what has been reported previously for mixed populations of pediatric and adult patients [3, 21]. This could be explained by a more subtle disease presentation in patients who survive into adulthood, leading to later onset and a longer delay in diagnosis. Studies reporting the prevalence of cognitive impairment in solely adult populations are lacking; however, only around one-quarter of the adult population in the present study had cognitive impairment at their latest visit, which is approximately 50% lower than what has been reported for the general MPS II population that includes children and adults [6, 7]. These data support previous findings showing that patients with non-neuronopathic MPS II survive longer into adulthood than patients with neuronopathic MPS II [8, 9]. Furthermore, awareness of MPS II has improved with time, and was likely lower when patients in this group were children and around the time they received a diagnosis, so it is possible that the early signs and symptoms were not considered to be related to MPS II and were not recorded appropriately.

Use of a hearing aid device was common in the analysis population (documented in 38.6% of treated patients), reflecting the fact that hearing loss is a common feature of MPS II [35]. Over 10% of hearing devices were put in place after the age of 18 years, highlighting the need for continued otolaryngological assessment in adult patients with MPS II.

It was previously reported that surgical interventions are common among patients with MPS II, particularly at a young age [28]. Our data showed that surgeries were also common in those surviving beyond the age of 18 years: most treated patients in the study population underwent one or two different surgical procedures beyond this point over a period of ~ 6 years. These findings suggest that many adults with MPS II remain at risk throughout their life of complications that require surgery, despite receiving ERT.

Consistent with previous findings from patients in HOS [28, 37], hernia repair was commonly reported for adult patients with MPS II, with over 12% of treated and untreated patients undergoing this surgery after the age of 18. Carpal tunnel decompression was also performed after the age of 18 in more than 10% of treated patients, highlighting the need to continue to screen for carpal tunnel syndrome into adulthood [37]. Monitoring for hernia or carpal tunnel syndrome recurrence after surgery is also recommended because surgeries for both conditions were common during childhood and frequently repeated in the treated adult population.

Treated patients with neuronopathic and non-neuronopathic MPS II underwent a similar number of different surgery types, as has been previously reported for patients in HOS [38]; however, the proportion of treated patients who required at least one surgery as an adult was higher in the non-neuronopathic group than in the neuronopathic group (54.3% vs. 29.5%, respectively). The reasons for this are unclear; however, hernia repair, carpal tunnel decompression, tracheotomy, and cervical decompression were carried out in a higher proportion of treated patients with non-neuronopathic than patients with neuronopathic MPS II. This could be due to the presence of more severe somatic disease manifestations in patients with neuronopathic MPS II, particularly in adulthood, which could lead to problems with anesthesia and could influence the decision to undergo surgery [23, 24]. Additionally, it may be more difficult to assess the need for carpal tunnel or cervical decompression surgery in patients with neuronopathic MPS II, owing to communication and cooperation difficulties [7]. By contrast, a higher proportion of patients with neuronopathic MPS II underwent gastrostomy/PEG tube insertion than patients with non-neuronopathic MPS II. This may suggest that cognitive impairment in adult patients with MPS II is associated with impaired eating and drinking, possibly as a result of dysphagia, which may increase the potential for aspiration or other difficulties.

Overall, our findings suggest differences between the surgical needs of patients with neuronopathic and non-neuronopathic MPS II during adulthood. However, these results should be interpreted in light of the following limitations. First, there were substantially more patients with non-neuronopathic versus neuronopathic disease in this analysis. The small sample size may partially explain why there were no recorded cases of tracheotomy, cervical decompression, hip replacement, adenoidectomy, intracranial shunt placement/replacement, or cervicolumbar fusion in the patients with neuronopathic MPS II. Second, patients with non-neuronopathic disease tended to be enrolled in HOS for a longer duration as adults than patients with neuronopathic disease, meaning that they spent more years at risk of surgeries or other complications. Finally, many patients received multiple types of surgery during childhood, which may have influenced their requirement for surgeries during adulthood.

Cardiovascular involvement has been reported to be common in patients with MPS II [8], supporting the finding that it was present in approximately three-quarters of patients in this study. The high prevalence of valve disease, which was observed in nearly 70% of adult patients, reflects similar findings for the general population of patients with MPS II [3]. Considering our finding that cardiovascular disease continues to be a leading cause of death in adult patients with MPS II, this highlights the need for cardiac monitoring and appropriate management of cardiovascular risk in this population.

Respiratory function was generally compromised in adult patients in HOS, with percentage of predicted FVC and FEV_1_ measurements below 85% and 75%, respectively, for most patients. Walking ability was also compromised in this patient population compared with the median 6MWT distance reported in healthy males (436 m versus 576 m) [39], and may have been influenced by the high incidence of cardiac and respiratory dysfunction, as well as joint involvement in this population. The high disease burden experienced by adults with MPS II in HOS highlights the need for psychological support for these patients because patients are likely to experience significant impacts on their abilities to perform daily activities and to function socially [40, 41].

Respiratory failure and cardiac arrest were previously reported to be the most common causes of death among patients of all ages in HOS [9, 21]. In this study of adult patients in HOS, respiratory failure, cardiac arrest, and cardiorespiratory failure were the first, second, and third most common known causes of death, respectively. This suggests that the main causes of death are broadly similar for pediatric and adult patients. However, causes of death are selected from a predefined list when recorded in HOS. This type of reporting provides only a partial clinical picture of the often complex and multisystemic factors that may contribute to respiratory failure or cardiac arrest in patients with MPS II.

ERT with idursulfase has been shown to stabilize or improve somatic signs and symptoms of MPS II [10–17]. In this study, percentage of predicted FVC was stabilized and uGAGs, 6MWT distance, and LVMI were improved between the first and the latest visits of patients in this study who had received at least one dose of idursulfase; however, percentage of predicted FEV_1_ decreased between first and latest visits, which may reflect the development, or increases in the severity, of obstructive pulmonary disease for individual patients. Overall, SAEs in the treated population occurred at a rate of three SAEs per 10 PY during adulthood. SAE rates were similar in treated adult patients with neuronopathic and non-neuronopathic disease, emphasizing the need for close monitoring of all treated adult patients with MPS II.

This study represents the first detailed analysis of the somatic disease burden and management of adult patients with MPS II, and utilizes the large patient population and wide range of data collected in HOS. However, as with other analyses of registry data, there are some limitations. These include difficulties associated with obtaining high‑quality, complete data for all patients. For example, concomitant medications and quality-of-life measures were of interest in this analysis, but these data are not captured systematically and are often incomplete in HOS. Another important consideration is that treated patients were defined as those who had received at least one dose of idursulfase and, as a result, some patients who were described as treated may have only received one, or a small number of, idursulfase doses. This, combined with the differences in ages at first and latest visits and at the start of idursulfase treatment, makes it difficult to analyze fully the effect of treatment in adults with MPS II; different methodologies would be needed to systematically assess the effects of ERT on the morbidity and mortality of adults with MPS II. Furthermore, cognitive impairment assessments used to classify patients into neuronopathic and non-neuronopathic groups could have been based on subjective assessment by the physician without formal cognitive testing, and the cognitive impairment status of patients was determined based on latest assessment only. Many patients may not fit the classical presentation of either neuronopathic or non-neuronopathic MPS II because cognitive impairment likely presents as a spectrum of severity rather than as two distinct disease states [38]. Indeed, just under one-third of the adult patients in the non-neuronopathic group were recorded as having cognitive impairment at a previous visit. These data illustrate the challenges that clinicians may face in identifying neuronopathic versus non-neuronopathic disease in adult patients with MPS II, particularly in the absence of formal cognitive testing. Finally, because methods and reference ranges for uGAG measurements differ between laboratories, the interpretation of these data may be limited.

## Conclusions

Overall, our findings demonstrate that adults with MPS II continue to experience a high disease burden and often require multiple surgeries, even while receiving disease-modifying ERT. Multidisciplinary management should continue throughout adulthood, including regular otolaryngological assessments, physical and radiological examinations including spinal assessments, and cardiac and respiratory assessments. Differences in the care needs of adult patients with neuronopathic and non-neuronopathic MPS II require further investigation, particularly as the lifespan of patients with MPS II continues to increase.

## Supplementary Information

**Additional file 1**. **Supplementary Table S1.** Patient characteristics stratified by treatment status (N = 373). **Supplementary Table S2.** Proportions of treated patients requiring repeat surgical procedures after 18 years of age. **Supplementary Table S3.** Proportions of adult patients that underwent surgical procedures before 18 years of age by treatment status. **Supplementary Table S4.** Clinical parameters in treated patients at last assessment by age at treatment start (N = 332). **Supplementary Table S5.** Clinical parameters for all patients at first and latest assessments (N = 373). **Supplementary Table S6.** Causes of death in treated patients (N = 332). **Supplementary Table S7.** Reclassification of surgeries originally listed as ‘other’ in the HOS database. **Supplementary Figure S1.** Age distribution for patients overall and for those with neuronopathic and nonneuronopathic disease at the latest visit. **Supplementary Figure S2.** Kaplan–Meier survival analysis from birth to date of death for treated adult patients (N = 332).

## Data Availability

The data sets, including the redacted study protocol, redacted statistical analysis plan, and individual participants’ data supporting the results reported in this article, will be made available within 3 months from initial request to researchers who provide a methodologically sound proposal. The data will be provided after its de-identification, in compliance with applicable privacy laws, data protection, and requirements for consent and anonymization.

## Abbreviations

6MWT: 6-Minute walk test
ERT: Enzyme replacement therapy
FEV_1_: Forced expiratory volume in 1 s
FVC: Forced vital capacity
GAG: Glycosaminoglycan
HOS: Hunter Outcome Survey
I2S: Iduronate‑2‑sulfatase
IRR: Infusion-related reaction
LVMI: Left ventricular mass index
MPS II: Mucopolysaccharidosis II
PEG: Percutaneous endoscopic gastrostomy
PY: Patient-years
SAE: Serious adverse event
uGAG: Urinary GAG

## References

[1] Neufeld EF, Muenzer J, et al. The Mucopolysaccharidoses. In: Scriver CR, Beaudet AL, Sly WS, et al., editors. The metabolic and molecular bases of inherited disease. New York: McGraw-Hill; 2001. p. 3421–52.

[2] Khan SA, Peracha H, Ballhausen D, et al. Epidemiology of mucopolysaccharidoses. Mol Genet Metab. 2017;121:227–40. 28595941 10.1016/j.ymgme.2017.05.016 PMC5653283

[3] Wraith JE, Beck M, Giugliani R, Clarke J, Martin R, Muenzer J. Initial report from the Hunter Outcome Survey. Genet Med. 2008;10:508–16. 18580692 10.1097/gim.0b013e31817701e6

[4] D’Avanzo F, Rigon L, Zanetti A, Tomanin R. Mucopolysaccharidosis type II: one hundred years of research, diagnosis, and treatment. Int J Mol Sci. 2020;21:1258. 32070051 10.3390/ijms21041258 PMC7072947

[5] Ayodele O, Muller K, Setayeshgar S, Alexanderian D, Yee KS. Clinical characteristics and healthcare resource utilization for patients with mucopolysaccharidosis II (MPS II) in the USA: a retrospective chart review. J Health Econ Outcomes Res. 2022;9:117–27. 35620452 10.36469/jheor.2022.33801 PMC9098230

[6] Young ID, Harper PS, Newcombe RG, Archer IM. A clinical and genetic study of Hunter’s syndrome. 2. Differences between the mild and severe forms. J Med Genet. 1982;19:408–11. 6818348 10.1136/jmg.19.6.408 PMC1048951

[7] Burton BK, Giugliani R. Diagnosing Hunter syndrome in pediatric practice: practical considerations and common pitfalls. Eur J Pediatr. 2012;171:631–9. 22383073 10.1007/s00431-012-1703-y PMC3306562

[8] Wraith JE, Scarpa M, Beck M, et al. Mucopolysaccharidosis type II (Hunter syndrome): a clinical review and recommendations for treatment in the era of enzyme replacement therapy. Eur J Pediatr. 2008;167:267–77. 18038146 10.1007/s00431-007-0635-4 PMC2234442

[9] Jones SA, Almassy Z, Beck M, et al. Mortality and cause of death in mucopolysaccharidosis type II-a historical review based on data from the Hunter Outcome Survey (HOS). J Inherit Metab Dis. 2009;32:534–43. 19597960 10.1007/s10545-009-1119-7

[10] Muenzer J, Wraith JE, Beck M, et al. A phase II/III clinical study of enzyme replacement therapy with idursulfase in mucopolysaccharidosis II (Hunter syndrome). Genet Med. 2006;8:465–73. 16912578 10.1097/01.gim.0000232477.37660.fb

[11] Muenzer J, Beck M, Eng CM, et al. Long-term, open-labeled extension study of idursulfase in the treatment of Hunter syndrome. Genet Med. 2011;13:95–101. 21150784 10.1097/GIM.0b013e3181fea459

[12] Dalmau Serra J, Vitoria Minana I, Calderon Fernandez R, Cortell AI. Clinical response to long term enzyme replacement treatment in children, adolescent and adult patients with Hunter syndrome. Med Clin (Barc). 2015;145:392–8. 26360015 10.1016/j.medcli.2015.06.015

[13] Lampe C, Bosserhoff A-K, Burton BK, et al. Long-term experience with enzyme replacement therapy (ERT) in MPS II patients with a severe phenotype: an international case series. J Inherit Metab Dis. 2014;37:823–9. 24596019 10.1007/s10545-014-9686-7 PMC4158409

[14] Parini R, Rigoldi M, Tedesco L, et al. Enzymatic replacement therapy for Hunter disease: up to 9 years experience with 17 patients. Mol Genet Metab Rep. 2015;3:65–74. 26937399 10.1016/j.ymgmr.2015.03.011 PMC4750582

[15] Tomanin R, Zanetti A, D’Avanzo F, et al. Clinical efficacy of enzyme replacement therapy in paediatric Hunter patients, an independent study of 3.5 years. Orphanet J Rare Dis. 2014;9:129. 25231261 10.1186/s13023-014-0129-1 PMC4180060

[16] Muenzer J, Giugliani R, Scarpa M, Tylki-Szymanska A, Jego V, Beck M. Clinical outcomes in idursulfase-treated patients with mucopolysaccharidosis type II: 3-year data from the hunter outcome survey (HOS). Orphanet J Rare Dis. 2017;12:161. 28974237 10.1186/s13023-017-0712-3 PMC5627440

[17] Okuyama T, Tanaka A, Suzuki Y, et al. Japan Elaprase Treatment (JET) study: idursulfase enzyme replacement therapy in adult patients with attenuated Hunter syndrome (Mucopolysaccharidosis II, MPS II). Mol Genet Metab. 2010;99:18–25. 19773189 10.1016/j.ymgme.2009.08.006

[18] Broomfield A, Davison J, Roberts J, et al. Ten years of enzyme replacement therapy in paediatric onset mucopolysaccharidosis II in England. Mol Genet Metab. 2020;129:98–105. 31383595 10.1016/j.ymgme.2019.07.016

[19] Whiteman DA, Kimura A. Development of idursulfase therapy for mucopolysaccharidosis type II (Hunter syndrome): the past, the present and the future. Drug Des Devel Ther. 2017;11:2467–80. 28860717 10.2147/DDDT.S139601 PMC5574592

[20] Garcia AR, DaCosta JM, Pan J, Muenzer J, Lamsa JC. Preclinical dose ranging studies for enzyme replacement therapy with idursulfase in a knock-out mouse model of MPS II. Mol Genet Metab. 2007;91:183–90. 17459751 10.1016/j.ymgme.2007.03.003

[21] Burton BK, Jego V, Mikl J, Jones SA. Survival in idursulfase-treated and untreated patients with mucopolysaccharidosis type II: data from the Hunter Outcome Survey (HOS). J Inherit Metab Dis. 2017;40:867–74. 28887757 10.1007/s10545-017-0075-x

[22] Marucha J, Jurecka A, Syczewska M, Rozdzynska-Swiatkowska A, Tylki-Szymanska A. Restricted joint range of motion in patients with MPS II: correlation with height, age and functional status. Acta Paediatr. 2012;101:e183–8. 22077147 10.1111/j.1651-2227.2011.02522.x

[23] Walker R, Belani KG, Braunlin EA, et al. Anaesthesia and airway management in mucopolysaccharidosis. J Inherit Metab Dis. 2013;36:211–9. 23197104 10.1007/s10545-012-9563-1 PMC3590422

[24] Suzuki K, Sakai H, Takahashi K. Perioperative airway management for aortic valve replacement in an adult with mucopolysaccharidosis type II (Hunter syndrome). JA Clin Rep. 2018;4:24. 29527552 10.1186/s40981-018-0162-5 PMC5838199

[25] Gadepalli C, Stepien KM, Sharma R, Jovanovic A, Tol G, Bentley A. Airway abnormalities in adult mucopolysaccharidosis and development of Salford Mucopolysaccharidosis Airway Score. J Clin Med. 2021;10:632. 34362059 10.3390/jcm10153275 PMC8347638

[26] Rutten M, Ciet P, van den Biggelaar R, et al. Severe tracheal and bronchial collapse in adults with type II mucopolysaccharidosis. Orphanet J Rare Dis. 2016;11:50. 27112191 10.1186/s13023-016-0425-z PMC4845328

[27] Lin HY, Chen MR, Lee CL, et al. Natural progression of cardiac features and long-term effects of enzyme replacement therapy in Taiwanese patients with mucopolysaccharidosis II. Orphanet J Rare Dis. 2021;16:99. 33622387 10.1186/s13023-021-01743-2 PMC7903682

[28] Mendelsohn NJ, Harmatz P, Bodamer O, et al. Importance of surgical history in diagnosing mucopolysaccharidosis type II (Hunter syndrome): data from the Hunter Outcome Survey. Genet Med. 2010;12:816–22. 21045710 10.1097/GIM.0b013e3181f6e74d

[29] Perez-Lopez J, Molto-Abad M, Munoz-Delgado C, Morales-Conejo M, Ceberio-Hualde L, Del Toro M. Efficacy of idursulfase therapy in patients with mucopolysaccharidosis type II who initiated enzyme replacement therapy in adult age. A systematic review of the literature. Mol Genet Metab. 2018;124:216–27. 29801985 10.1016/j.ymgme.2018.04.013

[30] Muenzer J, Jones SA, Tylki-Szymanska A, et al. Ten years of the Hunter Outcome Survey (HOS): insights, achievements, and lessons learned from a global patient registry. Orphanet J Rare Dis. 2017;12:82. 28464912 10.1186/s13023-017-0635-z PMC5414331

[31] Clark BM, Sprung J, Weingarten TN, Warner ME. Anesthesia for patients with mucopolysaccharidoses: comprehensive review of the literature with emphasis on airway management. Bosn J Basic Med Sci. 2018;18:1–7. 28590232 10.17305/bjbms.2017.2201 PMC5826667

[32] Hankinson JL, Odencrantz JR, Fedan KB. Spirometric reference values from a sample of the general U.S. population. Am J Respir Crit Care Med. 1999;159:179–87. 10.1164/ajrccm.159.1.9712108 9872837

[33] Polgar G, Promodhat V. Pulmonary function testing in children: techniques and standards. Philadelphia: W.B. Saunders; 1971.

[34] Devereux RB. Detection of left ventricular hypertrophy by M-mode echocardiography. Anatomic validation, standardization, and comparison to other methods. Hypertension. 1987;9:19–26. 10.1161/01.hyp.9.2_pt_2.ii19 2948914

[35] Keilmann A, Nakarat T, Bruce IA, Molter D, Malm G. HOS Investigators. Hearing loss in patients with mucopolysaccharidosis II: data from HOS-the Hunter Outcome Survey. J Inherit Metab Dis. 2012;35:343–53. 21866356 10.1007/s10545-011-9378-5

[36] Lau H, Harmatz P, Botha J, Audi J, Link B. Clinical characteristics and somatic burden of patients with mucopolysaccharidosis II with or without neurological involvement: an analysis from the Hunter Outcome Survey. Mol Genet Metab Rep. 2023;37:101005. 38053935 10.1016/j.ymgmr.2023.101005 PMC10694755

[37] Lin HY, Chuang CK, Chen MR, et al. Clinical characteristics and surgical history of Taiwanese patients with mucopolysaccharidosis type II: data from the hunter outcome survey (HOS). Orphanet J Rare Dis. 2018;13:89. 29866148 10.1186/s13023-018-0827-1 PMC5987665

[38] Link B, de Camargo Pinto LL, Giugliani R, et al. Orthopedic manifestations in patients with mucopolysaccharidosis type II (Hunter syndrome) enrolled in the Hunter Outcome Survey. Orthop Rev (Pavia). 2010;2:e16. 21808707 10.4081/or.2010.e16 PMC3143973

[39] Enright PL, Sherrill DL. Reference equations for the six-minute walk in healthy adults. Am J Respir Crit Care Med. 1998;158:1384–7. 9817683 10.1164/ajrccm.158.5.9710086

[40] Needham M, Packman W, Rappoport M, et al. MPS II: adaptive behavior of patients and impact on the family system. J Genet Couns. 2014;23:330–8. 24190099 10.1007/s10897-013-9665-4

[41] Needham M, Packman W, Quinn N, et al. Health-related quality of life in patients with MPS II. J Genet Couns. 2015;24:635–44. 25395377 10.1007/s10897-014-9791-7

