# Supplementary material for: Unmet needs of adults living with mucopolysaccharidosis II: data from the Hunter Outcome Survey
Source: Orphanet J Rare Dis. 2025 Jul 1;20:319. doi: 10.1186/s13023-024-03464-8 (PMC12211871; doi:10.1186/s13023-024-03464-8)
Supplement: Supplementary file 1 — Additional file 1. Supplementary Table S1. Patient characteristics stratified by treatment status (N = 373). Supplementary Table S2. Proportions of treated patients requiring repeat surgical procedures after 18 years of age. Supplementary Table S3. Proportions of adult patients that underwent surgical procedures before 18 years of age by treatment status. Supplementary Table S4. Clinical parameters in treated patients at last assessment by age at treatment start (N = 332). Supplementary Table S5. Clinical parameters for all patients at first and latest assessments (N = 373). Supplementary Table S6. Causes of death in treated patients (N = 332). Supplementary Table S7. Reclassification of surgeries originally listed as ‘other’ in the HOS database. Supplementary Figure S1. Age distribution for patients overall and for those with neuronopathic and nonneuronopathic disease at the latest visit. Supplementary Figure S2. Kaplan–Meier survival analysis from birth to date of death for treated adult patients (N = 332). [file 13023_2024_3464_MOESM1_ESM.pdf]

SUPPLEMENTARY MATERIALS

SUPPLEMENTARY TABLE S1 Patient characteristics stratified by treatment status (*N* = 373)

	**Treated patients (*n* = 332)**	**Untreated patients (*n* = 41)**	**All patients (*N* = 373)**
Age at first symptom onset, years	*n* = 279	*n* = 29	*n* = 308
Median (P10, P90)	2.0 (0.5, 6.0)	2.5 (0.2, 14.0)	2.0 (0.5, 6.0)
Age at diagnosis, years	*n* = 311	*n* = 37	*n* = 348
Median (P10, P90)	4.5 (1.7, 12.0)	5.5 (1.2, 35.1)	4.6 (1.5, 14.0)
Age at HOS entry, years	*n* = 332	*n* = 41	*n* = 373
Median (P10, P90)	16.7 (7.0, 32.1)	21.0 (12.6, 48.3)	17.3 (7.3, 33.2)
Time in HOS, years	*n* = 332	*n* = 41	*n* = 373
Median (P10, P90)	9.0 (1.5, 15.1)	2.2 (0.0, 8.4)	8.2 (0.8, 14.9)
Time in HOS as an adult, years	*n* = 332	*n* = 41	*n* = 373
Median (P10, P90)	6.1 (0.8, 20.5)	7.3 (0.6, 34.6)	6.2 (0.8, 21.7)
Age at latest visit, years	*n* = 332	*n* = 41	*n* = 373
Median (P10, P90)	24.1 (18.8, 38.5)	25.3 (18.6, 52.6)	24.2 (18.8, 39.7)
Deceased, *n* (%)	104 (31.3)	16 (39.0)	120 (32.2)
Age at death, years	*n* = 104	*n* = 16	*n* = 120
Median (P10, P90)	23.0 (18.8, 36.1)	21.8 (18.2, 52.6)	22.7 (18.7, 36.3)
Cognitive impairment, *n* (%)	*n* = 321	*n* = 36	*n* = 357
At any time	144 (44.9)	18 (50.0)	162 (45.4)
At latest visit	78 (24.3)	10 (27.8)	88 (24.6)

Abbreviations: HOS, Hunter Outcome Survey; P10, 10th percentile; P90, 90th percentile.

SUPPLEMENTARY TABLE S2 Proportions of treated patients requiring repeat surgical procedures after 18 years of age

	**Patients (*n* = 156)**	**Surgeries (*n* = 416)**
Any repeated surgery, n (%)	59 (37.8)	172 (41.3)
Hernia repair	21 (13.5)	51 (12.3)
Carpal tunnel decompression	10 (6.4)	26 (6.3)
Port-a-cath placement/replacement	11 (7.1)	26 (6.3)
Tracheotomy	6 (3.8)	13 (3.1)
Intracranial shunt placement/replacement	1 (0.6)	11 (2.6)
Gastrostomy/PEG tube insertion	3 (1.9)	10 (2.4)
Dental	3 (1.9)	8 (1.9)
Cervical decompression	3 (1.9)	6 (1.4)
Ear tube insertion	2 (1.3)	4 (1.0)
Valve replacement/repair	2 (1.3)	4 (1.0)
Hip osteotomy	1 (0.6)	3 (0.7)
Cervicolumbar fusion	1 (0.6)	2 (0.5)
Hip replacement	1 (0.6)	2 (0.5)
Other	3 (1.9)	6 (1.4)

Abbreviations: PEG, percutaneous endoscopic gastrostomy.

SUPPLEMENTARY TABLE S3 Proportions of adult patients that underwent surgical procedures before 18 years of age by treatment status

	**Treated patients (*n* = 332)**	**Untreated patients (*n* = 41)**	**All patients (*N* = 373)**
Any surgery, n (%)	257 (77.4)	21 (51.2)	278 (74.5)
Hernia repair	158 (47.6)	14 (34.1)	172 (46.1)
Ear tube insertion	146 (44.0)	6 (14.6)	152 (40.8)
Adenoidectomy	151 (45.5)	7 (17.1)	158 (42.4)
Tonsillectomy	113 (34.0)	3 (7.3)	116 (31.3)
Carpal tunnel decompression	96 (28.9)	0 (0.0)	96 (25.7)
Port-a-cath placement/replacement	62 (18.7)	0 (0.0)	62 (16.6)
Dental	47 (14.2)	2 (4.9)	49 (13.3)
Gastrostomy/PEG tube insertion	19 (5.7)	1 (2.4)	20 (5.4)
Intracranial shunt placement/replacement	18 (5.4)	3 (7.3)	21 (5.6)
Achilles lengthening	10 (3.0)	0 (0.0)	10 (2.7)
Tracheotomy	10 (3.0)	0 (0.0)	10 (2.7)
Trigger finger surgery	10 (3.0)	0 (0.0)	10 (2.7)
Cervical decompression	9 (2.7)	0 (0.0)	9 (2.4)
Femoral osteotomy	6 (1.8)	0 (0.0)	6 (1.6)
Genu varum	6 (1.8)	0 (0.0)	6 (1.6)
Valve replacement/repair	3 (0.9)	2 (4.9)	5 (1.3)
Cervical fusion	4 (1.2)	0 (0.0)	4 (1.1)
Cervicolumbar fusion	3 (0.9)	0 (0.0)	3 (0.8)
Hip replacement	3 (0.9)	0 (0.0)	3 (0.8)
Hip osteotomy	2 (0.6)	0 (0.0)	2 (0.5)
Knee arthroscopy	2 (0.6)	0 (0.0)	2 (0.5)
Unknown	1 (0.3)	0 (0.0)	1 (0.3)
Other	88 (26.5)	3 (7.3)	91 (24.4)

Abbreviations: PEG, percutaneous endoscopic gastrostomy.

SUPPLEMENTARY TABLE S4 Clinical parameters in treated patients at last assessment by age at treatment start (*N* = 332)

	**Age at treatment start**			**All treated patients (*N* = 332)**
	**0 to <9 years (*n* = 78)**	**≥9 to <18 years (*n* = 142)**	**≥18 years (*n* = 112)**	
uGAG	*n* = 2	*n* = 12	*n* = 14	*n* = 28
Mean (SD), µg/mg creatinine	43.2 (45.7)	87.4 (130.9)	42.5 (58.8)	61.8 (96.1)
Median (P10, P90), µg/mg creatinine	43.2 (10.9, 75.5)	46.4 (4.3, 198.2)	10.1 (4.5, 91.7)	4.3 (198.2)
Age at latest assessment, median (P10, P90) years	19.2 (18.6, 19.9)	21.1 (18.7, 23.5)	29.1 (22.4, 43.6)	23.4 (18.7, 41.7)
6MWT	*n* = 24	*n* = 62	*n* = 61	*n* = 147
Mean (SD), m	476.6 (125.8)	437.2 (172.3)	385.7 (138.4)	422.2 (154.6)
Median (P10, P90), m	476.5 (310.0, 648.0)	458.0 (255.0, 621.0)	400.0 (218.0, 526.0)	441.0 (221.0, 605.0)
Age at latest assessment, median (P10, P90) years	19.9 (18.3, 22.5)	23.1 (19.9, 30.0)	32.9 (24.5, 45.7)	26.2 (19.4, 39.7)
Any cardiovascular signs and symptoms				
n/N (%)	47/64 (73.4)	80/114 (70.2)	65/90 (72.2)	192/268 (71.6)
Valve disease				
n/N (%)	45/64 (70.3)	75/113 (66.4)	62/89 (69.7)	182/266 (68.4)
Cardiomyopathy	2/64 (3.1)	10/114 (8.8)	12/87 (13.8)	24/265 (9.1)

n/N (%)				
Arrhythmia/palpitations				
n/N (%)	2/62 (3.2)	11/111 (9.9)	9/84 (10.7)	22/257 (8.6)
LVMI	*n* = 24	*n* = 52	*n* = 52	*n* = 128
Mean (SD), g/m^2	87 (49.9)	95 (35.1)	103 (38.7)	97 (39.8)
Median (P10, P90), g/m^2	78 (57, 105)	85 (58, 137)	96 (62, 166)	88 (59, 151)
Age at latest assessment, median (P10, P90) years	19.9 (18.5, 23.2)	22.3 (18.9, 29.8)	32.0 (21.8, 47.0)	24.3 (18.9, 39.3)
Absolute FVC	*n* = 24	*n* = 68	*n* = 72	*n* = 164
Mean (SD), L	2.6 (1.2)	3.2 (6.7)	2.6 (3.1)	2.8 (4.8)
Median (P10, P90), L	2.3 (1.4, 4.4)	2.1 (1.0, 4.2)	2.3 (1.0, 3.6)	2.2 (1.1, 4.1)
Age at latest assessment, median (P10, P90) years	20.0 (18.3, 25.0)	22.5 (19.8, 30.8)	33.7 (25.3, 48.1)	27.1 (19.7, 41.9)
Percentage of predicted FVC	*n* = 25	*n* = 67	*n* = 70	*n* = 162
Mean (SD), %	63.8 (19.9)	58.5 (19.2)	61.1 (20.4)	60.4 (19.8)
Median (P10, P90), %	64.5 (39.7, 88.0)	57.0 (33.0, 83.0)	59.7 (36.0, 88.0)	59.4 (35.0, 85.3)
Age at latest assessment, median (P10, P90) years	19.9 (18.2, 25.0)	22.8 (19.5, 30.8)	33.8 (24.0, 48.8)	26.7 (19.5, 41.9)
Absolute FEV_1	*n* = 24	*n* = 68	*n* = 72	*n* = 164
Mean (SD), L	2.0 (1.0)	2.1 (3.2)	1.9 (2.7)	2.0 (2.7)
Median (P10, P90), L	1.9 (1.0, 3.5)	1.6 (0.6, 3.5)	1.4 (0.7, 2.9)	1.5 (0.6, 3.3)

Age at latest assessment, median (P10, P90) years	20.0 (18.3, 25.0)	22.5 (19.8, 30.8)	33.7 (25.3, 48.1)	27.1 (19.7, 41.9)
Percentage of predicted FEV_1	*n* = 25	*n* = 66	*n* = 70	*n* = 161
Mean (SD), %	55.3 (20.3)	47.1 (20.1)	46.8 (20.0)	48.2 (20.2)
Median (P10, P90), %	58.1 (31.0, 84.9)	47.5 (22.0, 71.0)	44.7 (24.0, 71.5)	46.8 (23.1, 74.7)
Age at latest assessment, median (P10, P90) years	19.9 (18.2, 25.0)	23.0 (19.8, 30.8)	33.8 (24.0, 48.8)	26.8 (19.6, 41.9)

Abbreviations: 6MWT, 6-minute walk test; FEV_1, forced expiratory volume in 1 second; FVC, forced vital capacity; LVMI, left ventricular mass index; m, meters; P10, 10th percentile; P90, 90th percentile; SD, standard deviation; uGAG, urinary glycosaminoglycan.

SUPPLEMENTARY TABLE S5 Clinical parameters for all patients at first and latest assessments (*N* = 373)

	**Neuronopathic (*n* = 88)**	**Non-neuronopathic (*n* = 269)**	**All patients (*N* = 373)[a]**
uGAG	*n* = 29	*n* = 90	*n* = 120
First visit, µg/mg creatinine	207.1 (55.0, 501.3)	176.0 (41.9, 490.1)	181.7 (44.5, 498.3)
Latest visit, µg/mg creatinine	73.9 (9.6, 250.0)	44.4 (13.0, 122.5)	46.0 (11.9, 152.2)
Time between first and latest visit, years	7.6 (1.4, 14.4)	6.7 (1.4, 14.1)	6.9 (1.4, 14.1)
6MWT	*n* = 14	*n* = 51	*n* = 65
First visit, m	343.5 (243.0, 429.0)	383.0 (274.0, 511.0)	375.0 (274.0, 503.0)
Latest visit, m	291.0 (109.0, 450.0)	413.0 (273.0, 526.0)	400.0 (252.0, 510.0)
Time between first and latest visit, years	7.8 (2.7, 15.1)	10.4 (2.4, 14.5)	9.5 (2.4, 15.1)
LVMI	*n* = 19	*n* = 49	*n* = 68
First visit, g/m^2	82.3 (51.6, 190.4)	95.7 (49.3, 185.5)	95.3 (49.9, 185.5)
Latest visit, g/m^2	77.4 (57.1, 105.5)	90.1 (60.6, 124.7)	83.8 (59.8, 120.5)
Time between first and latest visit, years	8.8 (1.8, 15.0)	8.8 (2.2, 13.8)	8.8 (2.0, 13.9)
Percentage of predicted FVC	*n* = 10	*n* = 51	*n* = 61
First visit, %	58.5 (44.4, 76.0)	70.0 (46.1, 89.0)	64.2 (44.8, 87.0)
Latest visit, %	53.5 (39.0, 59.1)	65.0 (34.5, 85.0)	61.0 (36.0, 83.0)
Time between first and latest visit, years	6.6 (2.0, 15.0)	6.9 (2.0, 13.9)	6.9 (2.0, 13.9)
Percentage of predicted FEV_1	*n* = 10	*n* = 52	*n* = 62
First visit, %	52.9 (38.5, 78.6)	61.0 (37.0, 86.0)	60.0 (38.0, 85.1)
Latest visit, %	46.0 (26.0, 88.2)	47.2 (25.6, 83.0)	47.2 (25.6, 83.0)
Time between first and latest visit, years	6.6 (2.0, 15.0)	7.0 (2.0, 13.9)	7.0 (2.0, 13.9)

All data are shown as median (P10, P90).

All patients were aged 18 years or older at their latest visit. However, baseline and/or latest visits for individual measurements may have taken place before patients reached 18 years of age.

[a]Cognitive data were not available for some patients; the number of patients in the 'all patients' population is therefore not equal to the combined total of the neuronopathic and non-neuronopathic MPS II populations.

Abbreviations: 6MWT, 6-minute walk test; FEV_1, forced expiratory volume in 1 second; FVC, forced vital capacity; LVMI, left ventricular mass index; m, meters; MPS II, mucopolysaccharidosis II; P10, 10th percentile; P90, 90th percentile; uGAG, urinary glycosaminoglycan.

SUPPLEMENTARY TABLE S6 Causes of death in treated patients (*N* = 332)

	**≥18 years to <28 years (*n* = 73)**	**≥28 years to <38 years (*n* = 23)**	**≥38 years (*n* = 8)**	**All patients (*n* = 104)**
Respiratory failure	19 (26.0)	8 (34.8)	2 (25.0)	29 (27.9)
Cardiac arrest	9 (12.3)	1 (4.3)	2 (25.0)	12 (11.5)
Cardiorespiratory failure	6 (8.2)	3 (13.0)	0 (0.0)	9 (8.7)
Pneumonia	4 (5.5)	2 (8.7)	0 (0.0)	6 (5.8)
Infection (sepsis)	4 (5.5)	2 (8.7)	0 (0.0)	6 (5.8)
Cardiac failure	4 (5.5)	0 (0.0)	0 (0.0)	4 (3.8)
Malignancy	0 (0.0)	1 (4.3)	0 (0.0)	1 (1.0)
Other	27 (37.0)	6 (26.1)	4 (50.0)	37 (35.6)

All data are *n* (%).

SUPPLEMENTARY TABLE S7 Reclassification of surgeries originally listed as 'other' in the HOS database

**New surgical category**	***n***
Achilles lengthening	9
Adenoidectomy	1
Cervical decompression	6
Cervicolumbar fusion	5
Dental	9
Ear tube insertion	5
Femoral osteotomy	3
Gastrostomy/PEG tube insertion	11
Genu varum	4
Hernia repair	2
Hip osteotomy	1
Intracranial shunt placement/replacement	11
Knee arthroscopy	2
Port-a-cath placement/replacement	47
Tonsillectomy	2
Tracheotomy	5
Valve replacement/repair	2
Unknown	1
**Total**	**130**

Abbreviations: HOS, Hunter Outcome Survey; PEG, percutaneous endoscopic gastrostomy.

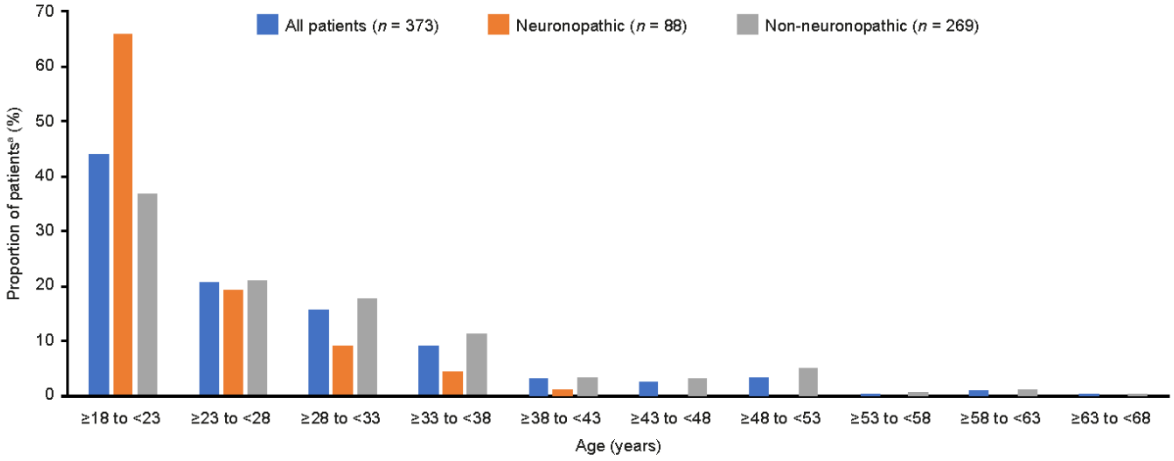

SUPPLEMENTARY FIGURE S1 Age distribution for patients overall and for those with neuronopathic and non-neuronopathic disease at the latest visit.

[a]Cognitive data were not available for some patients; the number of patients in the ‘all patients’ population is therefore not equal to the combined total of the neuronopathic and non-neuronopathic MPS II populations.

Abbreviations: MPS II, mucopolysaccharidosis II.

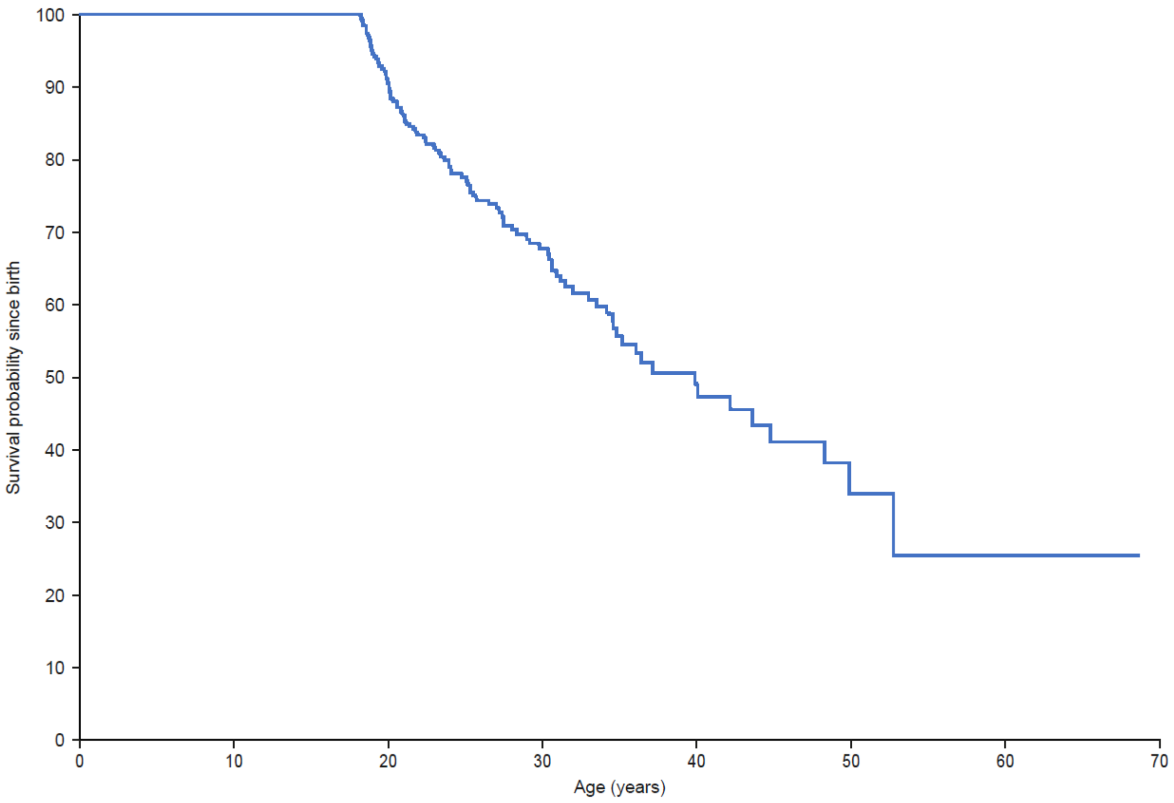

SUPPLEMENTARY FIGURE S2 Kaplan–Meier survival analysis from birth to date of death for treated adult patients (N = 332).

For patients who were alive at the time of this analysis, censoring was performed at the last-recorded study visit.
